# Draft Genome Sequence of *Natranaerobius trueperi* DSM 18760^T^, an Anaerobic, Halophilic, Alkaliphilic, Thermotolerant Bacterium Isolated from a Soda Lake

**DOI:** 10.1128/genomeA.00785-17

**Published:** 2017-09-07

**Authors:** Xiaomeng Guo, Ziya Liao, Mark Holtzapple, Qingping Hu, Baisuo Zhao

**Affiliations:** a College of Life Science, Shanxi Normal University, Linfen, Shanxi, People’s Republic of China; b Graduate School, Chinese Academy of Agricultural Sciences, Beijing, People’s Republic of China; c Department of Chemical Engineering, Texas A&M University, College Station, Texas, USA

## Abstract

The anaerobic, halophilic, alkaliphilic, thermotolerant bacterium *Natranaerobius trueperi* was isolated from a soda lake in Wadi An Natrun, Egypt. It grows optimally at 3.7 M Na^+^, pH 9.5, and 43°C. The draft genome consists of 2.63 Mb and is composed of 2,681 predicted genes. Genomic analysis showed that various genes are potentially involved in the adaptation mechanisms for osmotic stress, pH homeostasis, and high temperatures.

## GENOME ANNOUNCEMENT

The anaerobic, extremely halophilic, obligately alkaliphilic, and thermotolerant bacterium *Natranaerobius trueperi* was isolated from the sediments of an alkaline hypersaline lake in Wadi An Natrun, Egypt. In sodium bicarbonate-buffered medium (1), growth occurred at 3.1 to 5.4 M Na^+^ (optimum, 3.7 M Na^+^), pH 8.0 to 10.8 (optimum pH 9.5), and 26 to 52°C (optimum, 43°C) (our unpublished data). To understand the adaptive strategies for survival under multiple stress conditions, the genome sequencing of *N. trueperi* strain DSM 18760^T^ was performed using an Illumina HiSeq 4000 sequencer.

Genomic DNA extraction was performed using a microbial DNA isolation kit, according to the manufacturer’s instructions (New Industry, Beijing, China). A library for genome sequencing was constructed using the NEBNext Ultra DNA library prep kit for Illumina (2). Sequencing was carried out with a paired-end read length of 2 × 150 bp at approximate 200× coverage. The reads were *de novo* assembled using MicrobeTrakr plus version 0.9.1 (incorporates Velvet version 1.2.09). Quake and BWA were used in preassembly and postassembly sequence correction, respectively (3, 4). The total length of the draft genome sequence was 2,633,330 bp and yielded 127 contigs, with a GC content of 33.0% and an *N*_50_ value of 78,359 bp. Automated gene annotation was performed using the NCBI Prokaryotic Genome Annotation Pipeline (http://www.ncbi.nlm.nih.gov/genome/annotation_prok) and checked by the GenBank curation team. Among the predicted 2,681 genes, 2,588 putative protein-coding genes (CDSs) were identified. Furthermore, 93 RNAs, including 14 rRNAs (eight 5S RNAs, three 16S RNAs, and three 23S RNAs), 74 tRNAs, and 5 noncoding RNAs (ncRNAs) were found.

Genome sequence analysis of *N. trueperi* shed light on its strategies for surviving in a polyextreme environment, with elevated salinity and alkaline pH and moderately high temperature. The genome harbors 14 genes for glycine betaine ABC transporters, six genes for sodium-alanine symporters, three genes for sodium-proline symporters, two genes for sodium-solute symporters, and one gene for sodium-glutamate symporters, indicating that *N. trueperi* may resist osmotic stress at elevated salinity by taking up compatible solutes (i.e., betaine and amino acids) into cells from the extracellular environment (5, 6). Five genes (three TrkA-type and two TrkH-type) involved in the K^+^ uptake system also implied that *N. trueperi* possibly relies on salt (mainly K^+^) in cytoplasm to maintain osmotic balance at higher external salinity (5, 6). *N. trueperi* is obligately alkaliphilic and therefore must have an adaptive strategy for pH homeostasis (i.e., cytoplasmic acidification), because it possesses 8 genes of F1F0-ATP synthase (7), 7 genes of Na^+^/H^+^ antiporter (NhaC type), 4 genes of Na^+^ or K^+^/H^+^ antiporter (NhaP type), 2 genes of Na^+^/H^+^-dicarboxylate symporter (SDF family), and 1 gene of cation/H^+^ antiporter (CPA-2 family) (8). Also identified were 12 genes encoding orthologous rRNA methyltransferases (MTases) and 3 genes coding for orthologous tRNA MTases, which structurally stabilize DNA and RNA at moderately high temperature (9). One gene responsible for heat shock proteins was also found. These predicted genes on the genome of *N. trueperi* DSM 18760^T^ offer valuable insights to reveal the adaptation strategies of this polyextremophile.

### Accession number(s).

The draft genome assembly of *N. trueperi* DSM 18760^T^ has been deposited at DDBJ/ENA/GenBank under the accession number NIQC00000000.
